# Patient and carer feedback and engagement with ECHOMANTRA, a digital guided intervention for anorexia nervosa

**DOI:** 10.1002/erv.3140

**Published:** 2024-10-17

**Authors:** Valentina Cardi, Katie Rowlands, Suman Ambwani, Pamela Macdonald, Jon Arcelus, Ulrike Schmidt, Janet Treasure

**Affiliations:** ^1^ Department of General Psychology University of Padova Padova Italy; ^2^ Department of Psychological Medicine Centre for Research in Eating and Weight Disorders (CREW) Institute of Psychiatry Psychology and Neuroscience King's College London London UK; ^3^ DIS Study Abroad in Scandinavia Copenhagen Denmark; ^4^ Institute of Mental Health University of Nottingham Nottingham UK; ^5^ Bellvitge Biomedical Research Institute Hospitalet del Llobregat Barcelona Spain; ^6^ South London and Maudsley NHS Foundation Trust London UK

**Keywords:** carers, eating disorders, inpatient, online, self‐management

## Abstract

**Objective:**

We developed ECHOMANTRA, a digital guided intervention for patients with anorexia nervosa and their carers to provide support during transition from inpatient care to community settings. This study reports on participants' engagement with, and feedback of, ECHOMANTRA.

**Method:**

Patients and carers (*N* = 184 dyads) were given access to ECHOMANTRA for 12 months. The intervention included online groups, a workbook and recovery‐oriented videoclips. Satisfactory engagement was defined as attendance of a minimum of four online groups by each dyad. Participants received an Intervention Feedback Form to measure frequency of use and provide feedback of the intervention. Those who did not meet the engagement criterion were asked to complete an Obstacles to Engagement Form.

**Results:**

19% of the sample reached the engagement criterion. Seventy‐six patients and 60 carers completed the Intervention Feedback Form. Of those, approximately 60% reported using at least a quarter of the workbook and videoclips. Overall, participants found the materials useful and easy to access (median = 3 on a scale 1–5). Obstacles to engagement (35 patients and 14 carers) included lack of time due to caring responsibilities, treatment, work/school commitments.

**Conclusion:**

A more personalised form of support may be needed to enhance motivation and ability to change following inpatient care.

## Highlights

- ECHOMANTRA is a digital intervention to support after intensive care for anorexia nervosa
- 19% of patients and carers (total *N* = 184 dyads) met the satisfactory engagement criterion with ECHOMANTRA
- The most frequently reported obstacle to engagement was lack of time

## 1 INTRODUCTION

Approximately 20% of people with anorexia nervosa develop a severe and/or treatment refractory form of the illness that has a significant negative impact on health and quality of life (Reay et al., 2022). In such cases, inpatient or day patient care is needed to manage medical and mental health risks. However, the gains made during hospitalisation are often not sustained following discharge (Treasure et al., 2021). Relapse rates after inpatient treatment range from 20% to 50% (Berends et al., 2018), with most relapses occurring during the first 60 days after discharge (Walsh et al., 2021). Repeated admissions have increased at a faster rate than first recorded episodes (Holland et al., 2016) and mortality rates in the year following hospitalisation are increased 11.5 (95% confidence interval‐CI‐[6.0–17.0]) fold in those aged 15–24 and 14.0 (95% CI [9.2–18.8]) fold in those aged 25–44 years (Hoang et al., 2014). This evidence has led international experts to define current aftercare procedures as ‘inadequate’ (Bulik, 2021), feedback which has been echoed by patients and carers themselves (Clark Bryan et al., 2022). A further complication is that research into the management of patients with severe longstanding anorexia nervosa is not easy. For example, a recent trial to examine the relative effectiveness of inpatient care and a stepped care day patient approach was prematurely ended because of poor levels of recruitment (Philipps et al., 2023).

Two systematic reviews have examined the form and efficacy of aftercare interventions in eating disorders (Clark Bryan et al., 2021; Giel et al., 2021). The TIDIER framework, a template for intervention description and replication (Hoffmann et al., 2014) was used to describe the form and content of these interventions in 14 studies. The interventions included face‐to‐face support (*n* = 4), guided self‐help (*n* = 5), medication (i.e., fluoxetine, *n* = 3), indirect support to carers/family members (alone *n* = 1; joint carer/patient *n* = 2) and stepped‐care approaches (*n* = 2) (Clark Bryan et al., 2021). In a second review, seven randomised‐controlled trials (with three more registered) were examined (Giel et al., 2021). All studies, and particularly those that used pharmacological treatment, had high levels of drop out. Interventions which included bridging psychological support and/or increased social support from carers provided some benefit. However, most of these studies had low power, did not test efficacy, and were developed with limited input from patients and their families.

In 2017, based on a collaborative approach with patients and families and a series of proof of concept and feasibility studies (Cardi et al., 2019; Hibbs et al., 2015; Magill et al., 2016), our research group published the protocol of a fully powered randomised controlled trial (i.e., The Transition Care in Anorexia Nervosa Through Guidance Online from Peer and Carer Expertise, ‘TRIANGLE’ trial) aimed at testing ECHOMANTRA, a novel digital intervention for both patients with anorexia nervosa and their carers (Cardi et al., 2017). ECHOMANTRA targeted key intrapersonal and interpersonal maintaining factors of the illness, based on the premises of the cognitive interpersonal model of anorexia nervosa (Schmidt & Treasure, 2006; Treasure et al., 2020). The intervention for patients included some elements of the Maudsley Model of treatment for Anorexia Nervosa (MANTRA) (Schmidt et al., 2017) and had been previously adapted by including videos of recovery stories from people with lived experience (Cardi et al., 2019; Cardi e al., 2015). The carer intervention (i.e., Experienced Carers Helping Others: ECHO), was codeveloped with people with lived experience (Langley et al., 2018; Treasure et al., 2016) and had shown potential in previous proof of concept and feasibility studies (Hibbs et al., 2015; Magill et al., 2016).

The aims of the current study were to examine (1) participants' engagement with the ECHOMANTRA components (i.e., frequency of participation to the online groups, which was used to define overall engagement with the intervention materials, and frequency of use of videos and workbook), (2) participants' feedback about the components of the intervention (e.g., ease of access and usefulness), and (3) obstacles to engagement with the ECHOMANTRA materials.

## 2 METHODS

### 2.1 Study design

The published protocol describes the methods and materials of the TRIANGLE randomised controlled trial (Cardi et al., 2017). TRIANGLE tested the effectiveness of ECHOMANTRA, although findings related to the effectiveness of this intervention (which was added to treatment as usual (TAU) and compared to TAU alone) are reported elsewhere (Cardi et al., 2024). Recruitment occurred from April 2017 to June 2020, from 31 inpatient/day‐patient eating disorder services (independent and NHS) in the UK. Participants were approached by the clinical staff at the eating disorder services and if interested to take part in the study, they were then contacted by the researchers. The inclusion criteria were: patients aged 16 years or older, with a DSM‐5 diagnosis of anorexia nervosa, or atypical anorexia nervosa, receiving inpatient or intensive day care (minimum three days/week) who were able to nominate an informal supporter (family member or friend) willing to participate with them (i.e., defined as ‘carer’). Participants also needed access to an electronic device and the Internet during the course of the 18‐month study.

Following the completion of the baseline assessments, participants (the patient and carer dyad) were randomised with a ratio of 1:1 (using a minimisation algorithm to stratify by site and illness severity, defined by a body mass index <15 or >15 at baseline) to receive either (i) access to the ECHOMANTRA intervention in addition to TAU or (ii) TAU alone. Those allocated to the ECHOMANTRA intervention were required to use the intervention materials as often as they felt comfortable with, for 12 months following randomisation. The intervention components consisted of recovery‐oriented videoclips, a workbook and weekly online facilitated groups. The online facilitated groups were for patients only, carers only, or mixed, that is, for patients and carers together. Participants could attend more than a group/week if they decided to attend both a patient‐only or a carer‐only and a joint patient‐carer groups. Satisfactory engagement with ECHOMANTRA was established based on the attendance of the online groups (a minimum of four for both patients and carers). This is because ECHOMANTRA is based on a theoretical model where interpersonal relationships are of key importance to overcome the illness (Schmidt et al., 2019) and the online groups were the interactive part of this intervention. Furthermore, attendance of the online groups was automatically recorded by the study's platform and therefore could be considered a more accurate marker of engagement than participants' recall.

### 2.2 ECHOMANTRA

The ECHOMANTRA materials were accessed through an online platform developed for the trial by Mindwaves. They included weekly online facilitated and moderated groups (patients only, carers only, and patients and carers mixed groups), a video library (brief videos presenting lived‐experience recovery tips; role plays and professional psychoeducational tips), and written materials (patient and carer workbooks). Further information about the intervention, including training and supervision of the groups' facilitators are provided in Table 1.

**TABLE 1:** Template for Intervention Description and Replication (TIDierR) of the ECHOMANTRA intervention.

TIDieR items	Description of contents
Brief name of the intervention	ECHOMANTRA
Why: Rationale/goals	ECHOMANTRA is based on the cognitive interpersonal model of anorexia nervosa. The goal is to build collaboration between patients and carers and sustain therapeutic gains after hospital discharge. Patients receive the MANTRA materials, which are based on challenging the cognitive, emotional, interpersonal and nutritional obstacles to recovery. Carers receive the ECHO materials, in order to build confidence and skills in coping with the eating disorders thoughts and behaviours
What: Materials used	ECHOMANTRA consists of access to short video clips (recovery narratives by people with lived experience, psychoeducation from professionals or scenarios modelling behaviour change in the communication between patients and carers), a workbook and synchronous facilitated and moderated groups for patients only, carers only, or patients and carers together. These materials could be accessed through an encrypted and password‐protected website
What: Procedures, activities	Patients and carers could access the video clips and workbook at any given time, by connecting to the study's website, using their own password. Through the platform, they could also check the dates for the next online groups, book a place, and participate in the groups. The groups lasted approximately 60 min and were themed (over cycles of 8 sessions) and facilitated through the use of motivational interviewing techniques. Groups for patients or carers only and joint groups were held over alternate weeks
Who provided	The videoclips were recorded by people with lived experience of the illness (recovered individuals or carers) and professionals. The workbook was written by professionals and academics, in collaboration with people with lived experience of eating disorders. The groups were facilitated by the research assistants working on the trial, who had been trained in the use of motivational interviewing techniques and who were supervised weekly by clinical academics experts in eating disorders.
How: Mode of delivery	Participants could access the digital materials from randomisation up to 12 months after randomisation
Where: Location	Study's online platform
When and how much	Participants could access the workbook and videoclips through the online platform as often as they wished to. They could join the online groups weekly
Tailoring	No personalisation was used
Modifications	During the early phase of the study, the videoclips'library was extended by 13%. The workbook was not modified over the course of the trial
How well: planned engagement	Satisfactory engagement was defined as attendance of four online groups by both patients and carers
How well: actual engagement	19.46% of dyads participated in at least 4 online groups/each

### 2.3 Measures

#### 2.3.1 Sociodemographic and clinical information

Sociodemographic and clinical features (e.g., age, gender, recruitment site, carers' relationship to patient) were self‐reported through a survey completed on the study's website, prior to randomisation.

#### 2.3.2 Level of engagement with the online groups

The frequency of participation in the online groups (number and type of groups attended) was automatically recorded by the study's website.

#### 2.3.3 Level of engagement with the videos and the workbook

Frequencies of use for the workbook and videoclips were self‐reported through completion of an Intervention Feedback Form. The form could be completed by all participants randomised to ECHOMANTRA (*N* = 184) 12 months after randomisation, through the study's website. Questions included items such as ‘*What proportion of the video clips did you manage to watch?*’ and were answered on Likert scales ranging from 1 (e.g., ‘none’ or ‘very few’) to 5 (e.g., ‘all’ or ‘almost all’).

#### 2.3.4 Feedback on the intervention's materials

Feedback on the intervention materials could be provided by all participants randomised to ECHOMANTRA (*N* = 184), 12 months after randomisation and through the study website, using the Intervention Feedback Form. Questions investigated areas such as ease of access and usefulness of the resources and opinions about the characteristics of the groups' facilitator. Ratings were provided on likert scales ranging from 0 to 5 (i.e., higher numbers indicated a more positive opinion).

#### 2.3.5 Obstacles to engagement

Participants with limited engagement (i.e., those who had not reached the satisfactory engagement criterion by 12 months from randomisation), were invited to complete the Obstacles to Engagement Form. This form included stem questions, such as ‘What were the main obstacles for you to engage with the ECHOMANTRA intervention?’ and suggested answers such as ‘Lack of time due to commitments related to treatment…’ and ‘The content was unappealing…’. Each statement was rated on a Likert scale from 1 (never an obstacle) to 7 (always an obstacle). The questionnaire also included three free‐text items, for example, ‘Were there any other features (not listed above) that impacted your ability to engage with the materials? Please explain briefly’ (see Table 4).

### 2.4 Data analyses

Frequency of groups attendance and quantitative ratings from the Intervention Feedback and Obstacles to Engagement Forms were analysed using IBM SPSS Statistics Version 27. Data were described using medians and interquartile ranges. The free text completions about obstacles to engagement were analysed using thematic content analysis (Braun et al., 2016).

All participants in the trial who were randomised to ECHOMANTRA received the Intervention Feedback Form, whereas only a subgroup received the Obstacles to Engagement Form (i.e., only those who did not reach the satisfactory engagement criterion by 12 months). The number of participants completing one form and/or the other differed. Ratings of the Obstacles to Engagement Form were provided anonymously to enable participants to express their opinions freely. Due to anonymisation of these ratings, it was not possible to match data from the same participants, across the different questionnaires.

### 2.5 Definition of satisfactory engagement

The only pre‐specified satisfactory engagement criterion for both patients and carers in ECHOMANTRA was the attendance of a minimum of four online groups. No pre‐specified satisfactory engagement criteria were formulated for the use of the online video‐clips and workbook.

## 3 RESULTS

### 3.1 Sociodemographic and clinical features (*N* = 184 patient‐carer dyads)

One‐hundred and eighty‐five patient‐carer dyads were randomised to ECHOMANTRA. Demographic and clinical data were available for 184 dyads, because one dyad was randomised by mistake. Among patients, 92.9% were of female gender and 94.6% of White ethnic background. Their mean age was 25.8 years (SD = 9.4). The average duration of illness was 8.4 years (SD = 8.3); average height was 165.9 cm (SD = 7.7) and average self‐reported weight was 43.8 kg (SD = 6.9). Sixty‐three percent reported having a diagnosis of depression and 59.2% a diagnosis of anxiety disorders. The average age of carers was 49.9 years (SD = 12.6) and 69.6% were females. Most carers were parents (76.6%) and had a full‐time job (44.6%).

### 3.2 Attendance of the online groups: Recorded by the website (*n* = 184 dyads)

**FIGURE 1:**
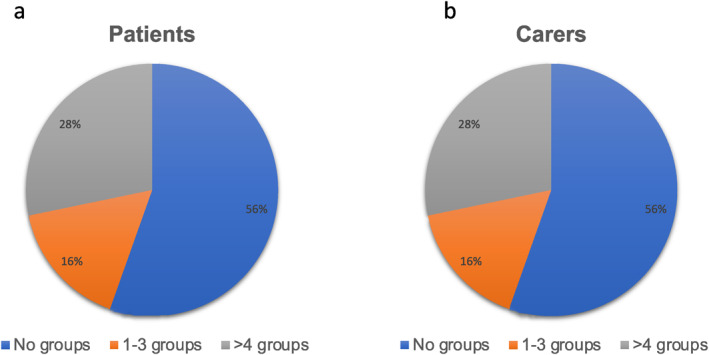
Percentages of patients (a) and carers (b) attending no groups, 1–3 groups or more than 4 groups.

The frequency of patients' and carers' participation in the online groups was recorded automatically for all participants randomised to ECHOMANTRA (*n* = 184) and is shown in Figure 1. Only 19% of patient‐carer dyads reached the pre‐set criterion of engagement, both attending a minimum of four online groups.

**FIGURE 2:**
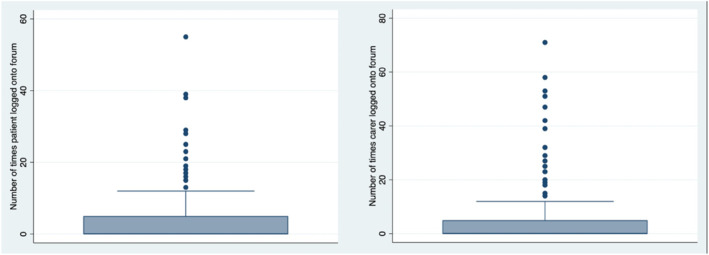
Number of times each patient within the ECHOMANTRA arm participated in an online group (left) and number of times each carer within the intervention arm participated in an online group (right).

The maximum number of groups attended by patients was 55 and the maximum attended by carers was 71 (Figure 2). Just over a half of both patients and carers (56%) failed to join any groups.

### 3.3 Intervention feedback form: Level of engagement

The Intervention Feedback Form was sent to 184 patient‐carer dyads and completed by 74 patients and 60 carers. Data from this form are described in Tables 2 and 3. With regards to engagement rates, approximately 60% of patients (59% of *N* = 74 patients) and carers (67% of *N* = 60 carers) reported that they had read at least a quarter of the workbook. Similar proportions (63% of *N* = 74 patients and 73% of *N* = 60 carers) reported that they had watched at least a quarter of all the videoclips in the library (Table 2).

**TABLE 2:** Participants' self‐reported engagement with the ECHOMANTRA workbook and videoclips.

	Proportion	Patients (*n* = 74) N (%)	Carers (*n* = 60) N (%)
Proportion of the workbook read	None/very little	30 (40.5%)	20 (33%)
	A quarter to half	25 (33.8%)	24 (40%)
	All or almost all	19 (25.7%)	16 (26.7%)
Proportion of videoclips watched	None/very little	27 (36.5%)	16 (26.7%)
	A quarter to half	33 (44.6%)	30 (50%)
	All or almost all	14 (18.9%)	14 (23.3%)

**TABLE 3:** Participants' self‐reported feedback of the ECHOMANTRA workbook, videoclips and online groups, and obstacles to engagement.

Intervention feedback form: Items	Patients (N = 74) Med (IQR)	Carers (N = 60) Med (IQR)
Easiness to access the videoclips	3 (3–4)	3 (3–4)
Easiness to access the online groups	3 (2–4)	3 (3–4)
Usefulness of information in the videoclips	3 (2–4)	3 (3–4)
Usefulness of information in the workbook	3 (2–3)	3 (3–4)
Usefulness of joining the online groups	3 (2–4)	3 (2–4)
Usefulness to read comments by other group members	3 (2–4)	3 (3–4)
Extent to which the group's facilitator seemed understanding	5 (4–5)	5 (4–5)
Extent to which the facilitator guided well the online groups	5 (4–5)	5 (4–5)
Extent to which participants looked forward to attending the groups	3 (2–4)	3 (2–4)
Obstacles to engagement form: Items	Patients (N = 35) Med (IQR)	Carers (N = 14) Med (IQR)
Lack of time due to treatment commitments	4 (3–5)	
Lack of time due to caring for the patient		4 (3–5.25)
Lack of time due to other caring responsibilities	2 (1–4)	4 (2.75–5)
Lack of time due to childcare difficulties	1 (1–1)	1 (1–1.50)
Lack of time due to work or study commitments	4 (3–5)	4 (1–5)
Intervention content not appealing	4 (2–5)	3 (1–4)
Intervention format not appealing	4 (2–4)	2 (1.75–3)
Access problems	1 (1–4)	2 (1–4)

*Note*: Data expressed as medians (Med) and inter‐quartile ranges (IQR). The Likert scale for the Intervention Feedback Form ranged from 1 ‘not at all’ to 5 ‘extremely’. The Likert scale for the Obstacles to Engagement Form ranged from 1 “never an obstacle to 7 ‘always an obstacle’.

With regards to the overall experience of ECHOMANRA, participants were asked questions about ease of access and usefulness of the materials and knowledge and skills of the groups' facilitators. Participants' scores are provided in Table 3. The most often reported score for ease of access and usefulness of the resources was three (on a scale ranging from 1 to 5). The highest median scores (score = 5) were provided for the quality of the guidance provided by the groups' facilitators.

### 3.4 Obstacles to Engagement Form

The Obstacles to Engagement Form was sent to 132 patient‐carer dyads who had not reached the engagement criterion by 12 months. The form was completed by 35 patients and 14 carers and data are described in Table 3. The difficulties most often reported with regards to the use of the intervention were related to lack of time due to treatment (patients) or caring (carers)’ responsibilities (median = 4 on a scale 1–7) and to work or school‐related commitments (median = 4 on a scale 1–7). For some patients, an obstacle to engagement was the modest appeal of the intervention's content and format (median = 4, on a scale 1–7).

The themes emerged from the thematic analysis of answers to the open questions are described in Table 4.

**TABLE 4:** Themes and subthemes emerged from the qualitative analysis of the Obstacles to Engagement Form.

Themes	Subthemes
Intervention contents	Emotionally challenging to engage with the content (P)
	Shame or denial about the eating disorder diagnosis (P)
	Fear of relapse from exposure to eating disorder contents (P)
Commitment	Need for more personalised check‐ins for engagement (P)
	Lack of time (P and C)
	Interpersonal challenges (P and C)
Need for individualisation	Lack of diversity of contents (P)
	Contents not tailored to different stages of recovery (P)
Digital format of the intervention	Technical challenges (P and C)
	No face‐to‐face contact (P and C)
	Difficulties to build trust (P)

*Note*: Obstacles reported by patients are identified with ‘P’. Obstacles reported by carers are identified with ‘C’.

## 4 DISCUSSION

The aim of this paper was to describe participant engagement with and feedback regarding ECHOMANTRA, a digital, guided self‐management intervention for patients with anorexia nervosa and their carers. Only 19% of patients and carers (out of 184 dyads) met the intervention satisfactory engagement criterion, however of those who completed the Intervention Feedback Form (76 patients and 60 carers), approximately 60% reported having used at least a quarter of the videoclips and workbook. Participants rated the digital resources as overall useful and easy to access and provided the highest scores of acceptability for the online groups' facilitators.

The suboptimal engagement with the ECHOMANTRA materials is consistent with the broader literature on usage of digital interventions for people with mental health disorders. For example, a systematic review of user engagement with online programs for depression or anxiety in the real‐world found that between 21% and 88% of users engaged in minimal use (e.g., intervention used at least once) and that around 7%–42% completed between 40% and 60% of the program (defined as ‘moderate use’; Fleming et al., 2018). Completion of the program or sustained use (use of the program for six or more weeks) had significantly smaller percentages, ranging from 0.5% to 28.6% (Fleming et al., 2018). Even when digital interventions are delivered with human support, the extent to which this might enhance engagement varies greatly between studies and is very uncertain (Lipschitz et al., 2023).

The finding of suboptimal engagement with the online groups aligns also with previous work in the eating disorder literature, which has found that patients with a severe and enduring form of anorexia nervosa are challenging to recruit and retain in treatment (Wonderlich et al., 2020). A systematic review concluded that approximately 40% of people receiving psychological treatments for an eating disorder do not complete the full course of treatment. The withdrawal rates range from 4% for family therapy in adolescents and to up to 100% for dietary advice (DeJong, Broadbent, and Schmidt, 2012). A recent longitudinal outpatient study from Norway reported that 69% of patients with anorexia nervosa prematurely terminated CBT‐E (one of the most widely recommended treatments; Kessler et al., 2022). Patients' explanations for premature termination of eating disorder treatments were summarised in a systematic review and qualitative synthesis (Vinchenzo et al., 2022). Participants explained that their ambivalence about change was because the eating disorder had become enmeshed with their identity and they did not have enough support to reconstruct a sense of self without the illness. They reported feeling misunderstood because of this failure to have a shared goal. Patients in the TRIANGLE trial had moderate levels of motivation when recruited but this fell over time (baseline median = 8, 12‐month median = 7; Cardi et al., personal communication). Self‐reported ability to change remained stable, and low (baseline median = 3, 12‐month median = 3). In the current study, additional reasons for suboptimal engagement with the ECHOMANTRA intervention included lack of time due to other treatment’ commitments and school‐related duties. Furthermore, the qualitative feedback highlighted reasons, such as lack of personalisation of the resources (e.g., materials targeted to different stages of the illness and different symptom presentations), the emotional challenges associated with facing illness‐related contents and uneasiness around the use of ‘at distance’ tools.

The involvement of carers is usually associated with better outcome from treatment (Adamson et al., 2019; Monteleone et al., 2022). Indeed, a slight higher proportion of carers than patients engaged with the written and video materials. However, there was limited involvement in the groups due to caring responsibilities and work‐related committments.

### 4.1 Limitations

A limitation of the current study is that it was not possible to implement an automatic system to record watching of the videoclips and access to the written workbook, and the self‐reports were completed only by a subgroup of participants (74/184 patients and 60/184 carers). Furthermore, the feedback forms were given after 12 months when almost 40% of participants (36.75%) had left the study. Earlier and more regular assessments of participants' engagement and feedback could provide helpful information and suggest strategies to overcome obstacles and sustain motivation. Another limitation in the design of this study is that only one carer was included with each participant. This may have inadvertently led to fragmentation within the informal systems of social support and exerted an adverse impact on the outcome (Treasure et al., 2015). Future studies would benefit from structured interviews to collect in‐depth information to test some of these hypotheses and improve the understanding of participants' difficulties with digital interventions.

Finally, it was not possible to integrate the components of ECHOMANTRA with the usual aftercare clinical management. Participants were recruited from specialised services commissioned across England and Scotland (both independent and NHS) and a lack of resources within local services limited the possibility for engagement and integration. Embedding ECHOMANTRA within usual forms of aftercare treatment might have shown greater uptake, engagement and benefits.

## 5 CONCLUSION

ECHOMANTRA is a digital guided self‐management intervention developed for patients with anorexia nervosa and their carers. The limited engagement with the interactive (group) component of the intervention might be underpinned by the low rating of ability to change even after a period of intensive care (Cardi et al., personal communication) and by patients' ambivalence to change. Many of these patients had an enduring illness and so had lost a critical phase of adolescent social and cognitive development. It is possible that in order to develop a recovered identity they may need a longer period of rehabilitation and support following intensive care rather than a repeated cycle of admissions that unfortunately often occurs. Families struggle with caregiving burden and life commitments and might lack time and resources to engage with additional support.

This study highlights the need to continue to engage with patients and carers in further regular consultations to develop personalised care pathways through the implementation of support strategies that are targeted to varied needs and stages of change.

## CONFLICT OF INTEREST STATEMENT

No conflicts of interest to report.

## PATIENT CONSENT STATEMENT

All participants provided written informed consent prior to participation.

## CLINICAL TRIAL REGISTRATION

The trial was pre‐registered on the ISRCTN registry (ISRCTN14644379) and the information are available at the following link https://doi.org/10.1186/ISRCTN14644379.

## Data Availability

Data are available on request to the corresponding author.
